# Parastomal hernia repair with onlay mesh remains a safe and effective approach

**DOI:** 10.1186/s12893-020-00964-9

**Published:** 2020-11-24

**Authors:** Marie Shella De Robles, Christopher J. Young

**Affiliations:** 1 Department of Colorectal Surgery, Royal Prince Alfred Hospital Medical Centre, Suite G07/100 Carillon Avenue, Newtown, Sydney, NSW 2042 Australia; 2 Discipline of Surgery, The University of Sydney, Sydney, NSW Australia

**Keywords:** Parastomal hernia, Mesh repair, Peristomal incision, Stoma relocation

## Abstract

**Background:**

Parastomal hernia (PSH) management poses difficulties due to significant rates of recurrence and morbidity after repair. This study aims to describe a practical approach for PSH, particularly with onlay mesh repair using a lateral peristomal incision.

**Methods:**

This is a retrospective review of consecutive patients who underwent PSH repair between 2001 and 2018.

**Results:**

Seventy-six consecutive PSH with a mean follow-up of 93.1 months were reviewed. Repair was carried out for end colostomy (40%), end ileostomy (25%), ileal conduit (21%), loop colostomy (6.5%) end-loop colostomy (5%) and loop ileostomy (2.5%). The repair was performed either with a lateral peristomal incision (59%) or a midline incision (41%). Polypropylene mesh (86%), biologic mesh (8%) and composite mesh (6%) were used. Stoma relocation was done in 9 patients (12%). Eight patients (11%) developed postoperative wound complications. Recurrence occurred in 16 patients (21%) with a mean time to recurrence at 29.4 months. No significant difference in wound complication and recurrence was observed based on the type of stoma, incision used, type of mesh used, and whether or not the stoma was repaired on the same site or relocated.

**Conclusion:**

Onlay mesh repair of PSH remains a practical and safe approach and could be an advantageous technique for high-risk patients. It can be performed using a lateral peristomal incision with low morbidity and an acceptable recurrence rate. However, for patients with significant adhesions and very large PSH, a midline approach with stoma relocation may also be considered.

## Background

Parastomal hernia (PSH) is a common complication of stoma formation in colorectal surgery, with an incidence up to 50% [1–3]. The risk of PSH is highest within the first few years after the formation of the stoma but may develop more than 40 years later. The incidence of PSH depends upon the length of follow-up and diagnostic criteria used—clinical, radiological or intraoperative findings. The lack of a standard definition along with minimal physical exam findings observed with some PSH occurrences make the actual incidence difficult to ascertain. PSH rates may increase with prolonged follow-up. Identified risk factors for PSH include advanced age, obesity, immunosuppression, increased intraabdominal pressure and post-operative wound infection [4].

PSHs are often asymptomatic and can be managed with conservative treatment. However, 11–70% of patients undergo surgery due to increasing hernia size, problems with the stomal appliance, discomfort, pain, and cosmetic dissatisfaction [2]. These treatment percentages vary significantly because surgeons are often reluctant to repair a PSH due to the high recurrence rate, complicated operation, and comorbidity of patients [2, 3]. The recurrence rate of PSH is lowest after mesh repair (0–33%), whereas primary fascial closure (46–100%) and relocation of the stoma (0–76%) result in much higher rates [1, 5–8]. Available studies show a broad range of reported parastomal hernia recurrence rates and no difference in mesh type concerning surgical site infection and hernia recurrence [5–8]. At present, no definite answer can be given as to whether there is a significant difference between the outcomes of synthetic and biologic mesh repair.

Stoma relocation may be required in some patients with very large PSH and with significant intra-abdominal adhesions. This approach entails a midline laparotomy which may be associated with higher morbidity. Unfortunately, not all patients can tolerate this major operative procedure [9]. Hence, this may not be an option for some patients. PSH repair without laparotomy is associated with a significantly shorter hospital stay, possibly due to the lack of a midline abdominal wound [10].

This study describes a practical approach for PSH, primarily with synthetic onlay mesh repair using a lateral peristomal incision. Herein, we evaluate the outcome of this technique with particular attention to patient safety, major mesh-related complication, and PSH recurrence.

## Methods

### Study design and participants

This is a retrospective cohort study of 76 consecutive cases of PSH managed by a single surgeon between February 2000 and February 2018. Data was collected for age, gender, type of stoma, the method of repair, type of incision and mesh used, Patients' data were obtained from their hospital records and the consulting surgeon's office.

### Outcomes of interest

The primary outcomes of interest were major mesh-related complications and PSH recurrence. Mesh-related complications were defined as any significant complication related to or caused by the presence of mesh, including mesh infection, bowel erosion or obstruction. PSH recurrence was defined as a bulge or fascial defect around the stoma site or midline, detected either by physical examination by a surgeon, or imaging studies done at any point in the postoperative period.

### Surgical technique

**Fig. 1:**
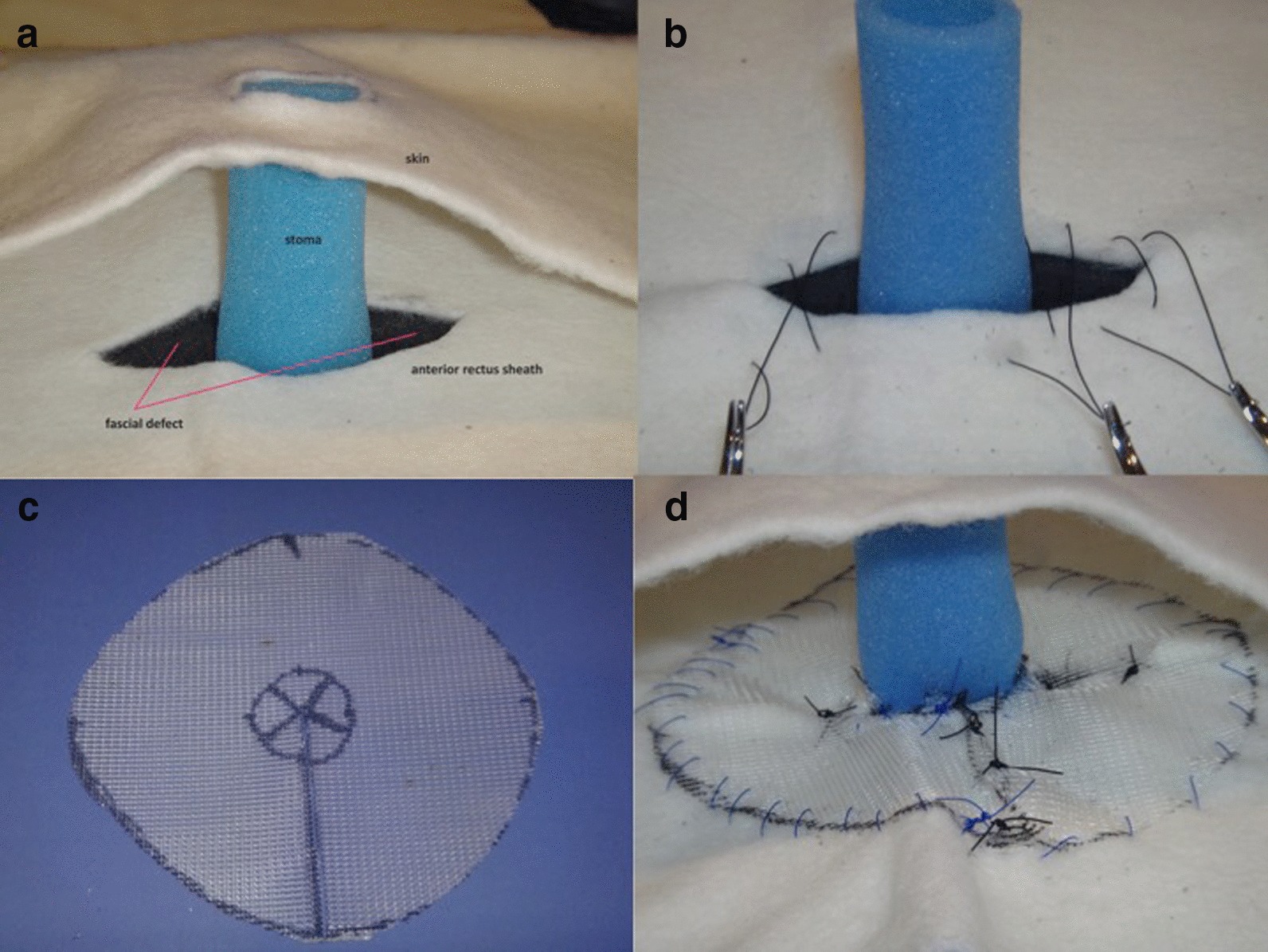
**a** Model of a parastomal hernia dissected down to the rectus sheath with contents reduced back inside the abdomen. The fascial defect can be readily appreciated; **b** The fascial defect closed with nonabsorbable suture, maintaining care to avoid narrowing the orifice too much; **c** The mesh should be cut in such a way that the mesh edge does not potentially erode into the bowel wall; **d** The mesh placed anterior to the anterior rectus sheath, sutures passed through the mesh and tied

Two types of surgical operations were performed to repair the PSH. The repair was done either with stoma relocation with a new stoma created under laparotomy at a different site and closure of the original stoma site, or direct repair by reinforcement with mesh after suture repair of the fascial defect. The later was done either with a midline or lateral peristomal incision. In the peristomal approach, a lateral incision approximately 10 cm away from the stoma was made, dissection was carried down to the anterior rectus sheath. Dissection should be continued until the hernia sac is isolated and can easily be reduced back into the abdomen (Fig. 1a). The fascial defect was closed with non-absorbable suture just enough to narrow the orifice (Fig. 1b). The repair was then reinforced with mesh which is fixed to the anterior fascia with non-absorbable suture (Fig. 1c). Mesh used to repair the PSH was either a polypropylene, composite or biologic mesh. Crucial to the mesh placement is to make sure that the mesh does not form a circle around the bowel, where the edge of the mesh is in direct contact with the bowel, and could potentially saw and erode into the bowel wall (Fig. 1d).

### Statistical analysis

The Pearson Chi and Fisher’s exact tests were used to test for significance of differences between groups. All analyses were conducted using SPSS version 23 (SPSS Inc., Chicago, IL, USA), and p < 0.05 was considered statistically significant.

## Results

The demographic details are shown in Table 1. Between February 2001 and February 2018, we treated 76 patients diagnosed with symptomatic PSH. The main presenting problem of the patients includes increasing hernia size, intermittent bowel obstruction, poor fit of the stomal appliance, pain, and discomfort.

**Table 1:** Demographic characteristics of patients undergoing parastomal hernia repair (n = 76)

Age (years)	
Mean + SD; range	63.0 ± 13.6; 32–83
Sex	
Male	38 (50%)
Female	38 (50%)
Stoma type	
End colostomy	30 (40%)
End ileostomy	19 (25%)
Ileal conduit	16 (21%)
Loop colostomy	5 (6.5%)
End-loop colostomy	4 (5%)
Loop ileostomy	2 (2.5%)
Incision, n = 69	
Peristomal	41 (59%)
Midline	28 (41%)
Mesh used, n = 64	
Polypropylene mesh	55 (86%)
Biologic mesh	5 (8%)
Composite mesh	4 (6%)
Complications	
Wound problem	8 (11%)
Recurrence	16 (21%)
Follow-up time (months)	
Mean + SD; range	93.1 ± 57.8; 3–218
Time to recurrence (months)	
Mean + SD; range	29.4 ± 20.7; 5–75
Operative time (h)	
Mean + SD; range	3.0 ± 1.7; 0.5–7.3
Length of hospital stay (days)	
Mean + SD; range	23.3 ± 37.9; 2–191

After a mean follow-up of 93.1 months (range 3–218; SD 57.8), PSH recurrence was noted in 16 patients (21%). The mean time to recurrence was 29.4 months (range 5–75, SD 20.7). Eight patients (11%) developed wound complications post-operatively. Two patients developed wound abscess that required drainage in theater, two other patients developed wound cellulitis that required antibiotic therapy, one had an infected polypropylene mesh that required removal, one had minimal wound breakdown that needed daily dressing, and one patient developed ischaemia of the skin and fat around the stoma that required debridement and resiting of colostomy. There were no cases of mesh erosion into the bowel. There were no deaths or major cardiopulmonary complications.

The overall mean and median length of hospital stay was 23.3 days (range 2–191, SD 37.9) and 10.5 days respectively. There was no significant difference in length of hospital stay regardless of the incision used (p = 0.365) and whether or not the stoma was repaired on the same site or relocated (p = 0.797).

Table 2 summarizes clinical outcomes after PSH repair. There was no significant difference in wound complications and hernia recurrence irrespective of the stoma type, incision used in PSH repair, type of mesh used, and whether or not the stoma was repaired on the same site or relocated.

**Table 2:** Comparison of wound complications and hernia recurrence after parastomal hernia repair

	Wound problem (N = 8)		Recurrence (N = 16)	
Stoma type				
Ileal conduit	3 (38%)		5 (31%)	
Loop ileostomy	0	p = 0.716	0	p = 0.511
End ileostomy	1 (13%)		5 (31%)	
Loop colostomy	1 (13%)		0	
End colostomy	3 (38%)		6 (38%)	
Abcarian colostomy	0		0	
Incision				
Peristomal	6 (75%)	p = 0.458	12 (75%)	p = 0.245
Midline	2 (25%)		4 (25%)	
Type of repair				
Direct repair	7 (88%)	p = 1.000	16 (100%)	p = 0.104
Stoma relocation	1 (13%)		0	
Mesh used				
Polypropylene	6 (75%)	p = 0.050	15 (94%)	p = 0.460
Composite	2 (25%)		0	
Biologic	0		1 (6%)	

## Discussion

Management of PSH can pose difficulties due to significant rates of recurrence and morbidities of the repair. The current standard of care is mesh repair whenever possible. Although low recurrence rates are reported after synthetic mesh repair, concerns have been raised regarding the safety of synthetic meshes in potentially contaminated fields due to the risk of mesh infection and subsequent removal. Other mesh-related complications include chronic infection, bowel stenosis, erosion of the mesh through the bowel and skin and entero-atmospheric fistulization [4, 11, 12]. These complications led to the development of biologic mesh, which due to its biodegradable nature, has the potential to improve these problems in infected and contaminated fields. The biologic mesh was then introduced for these cases and has become a popular choice for the past few years. Although a promising option, its popularity seized because of a few studies that showed a recurrence rate as high as 31%, when biologic mesh was used in a contaminated field during hernia repair [12]. Recently, the concept of avoiding a synthetic mesh in contaminated cases has been challenged. For instance, Carbonell et al. were able to show acceptable rates of surgical site infection when the synthetic mesh is used in clean-contaminated and contaminated hernia repairs [13]. Synthetic meshes, when used for either an open onlay or retromuscular repair, resulted in low surgical site infections and parastomal hernia recurrence rates [4, 11–14]. Our study showed no statistical difference in wound complication and hernia recurrence regardless of the type of mesh used.

In a small PSH where a small fascial defect leads to the accumulation of bowel and omentum in a subcutaneous pocket, hernia repair can be often accomplished by a direct surgical approach on the problem. However, patients with very large and recurrent PSH tend to have a greater fascial defect that can only be closed under considerable tension. Stoma relocation involves repositioning the stoma to a new location. At the same time, it involves fixing the hernia at the previous stoma site. Unfortunately, in patients with a history of PSH in the past, there is a significant risk of developing a PSH at the new stoma site. The reported risk was as high as 76% [4]. Similarly, this method also is associated with a risk of hernia recurrence as high as 52% at the previous stoma location [10]. Stoma relocation is usually a last resort when the existing stoma location is substandard and under a lot of tension. If this is the case, then the new stoma should be ideally placed on the opposite side of the abdomen because of the higher reported recurrence rates when the same side is used. Some also recommend using mesh to cover the old and new stoma sites as well as the midline incision to prevent a hernia [12].

In a study by Riansuwan et al. comparing outcomes after repair of recurrent PSHs between direct repair and stoma relocation, the later was associated with longer operative time and hospital stay [15]. Although they were able to show that the recurrence rate was initially lower in stoma relocation, calculated and longer predicted follow-up time did not show any difference in recurrent rates. This was supported by another study conducted by Baig which demonstrated that stoma relocation with a midline laparotomy was associated with longer operative time and hospital stay and morbidity rates [10]. The complications mentioned in the midline laparotomy were usually related to exposure of the hernia defect, difficulties dissecting dense adhesions caused by prior surgery and enterotomies incurred as a result of adhesiolysis. However, they also found no significant difference regarding recurrence rates [10]. This finding is consistent with our results. Similarly, our study was not able to demonstrate any significant difference regarding wound complication and hernia recurrence rates when direct mesh repair was compared to stoma relocation.

The onlay method of repair has been associated with low morbidity particularly regarding wound and mesh infection rates—1.9% and 2.6% respectively [16]. With this technique, the overall one-year recurrence rate was 17.2% (range 0–20%) [16]. More importantly, the advantage of the onlay repair is the avoidance of performing a laparotomy. This will not only be beneficial to high-risk patients with significant cardiopulmonary comorbidities, but also to patients with substantial intra-abdominal adhesions. Because of these reasons, this technique was utilized for all our patients who underwent mesh repair of the PSH using the lateral peristomal incision.

The onlay method of repair requires extensive skin mobilization to make room for the mesh. This creates a significant dead space, which can result in a seroma formation and ultimately may pose a potential risk for wound and mesh infections. While some view this as a significant disadvantage with the onlay technique, we did not have a substantial problem with this. Our study showed that lateral peristomal incision was associated with shorter operative time and length of hospital stay. However, there was no significant difference regarding wound complications and hernia recurrence when lateral peristomal incision and midline incision were compared.

Interpretation of the results was limited by the retrospective design of the study and the limited number of patients. A larger cohort of patients with longer follow-up and evaluated in a randomized, controlled trial would strengthen the results of this study.

## Conclusion

Our findings further reveal that mesh type was not associated with any difference in outcomes. Suturing of the fascial defect and the use of synthetic onlay mesh can be performed with minimal wound-related morbidity and similar recurrence rates. However, a midline approach with stoma relocation in selected patients may also be considered, particularly for those patients with significant adhesions and very large PSH. Overall, direct mesh repair of PSH using the lateral peristomal incision remains a practical and safe approach and could be an advantageous technique in selected patients.

## Data Availability

The datasets used and/or analysed during the current study are available from the corresponding author on reasonable request.

## Abbreviation

PSH: Parastomal hernia
